# Vertical scapular osteotomy in congenital high scapula

**DOI:** 10.1007/s11832-015-0676-6

**Published:** 2015-08-19

**Authors:** Tarek Hassan Abdelaziz, Shady Samir

**Affiliations:** Department of Orthopaedic Surgery, Ain Shams University, Cairo, Egypt

**Keywords:** Congenital high scapula, Vertical scapular osteotomy, Sprengel’s deformity

## Abstract

**Purpose:**

Children with congenital high scapula (CHS) have a cosmetic and functional problem due to limited shoulder abduction. Treatment options include excision of the prominent superior angle, scapular relocation procedures and subtotal scapulectomy. Excision of the superomedial angle results only in cosmetic improvement. Subtotal scapulectomy and relocation procedures are associated with ugly scars, extensive bleeding and high incidence of brachial plexus injuries. Vertical scapular osteotomy (VSO) is another surgical option that provides cosmetic and functional improvement. The aim of this study is to assess medium to long term results of VSO in treatment of CHS.

**Methods:**

This is a prospective case series study. Seven children with CHS were treated at our unit. Age ranged from 5–13 years with an average of 8.4 years. All children were females with unilateral affection. All children underwent a VSO as described by Campbell. We used the Cavendish grading system together with combined shoulder abduction for assessment. Follow up averaged 4.6 years.

**Results:**

All children and parents were extremely satisfied with the results of surgery. All patients experienced an improvement in global shoulder abduction with an average gain in abduction of 52.9°. All patients experienced an improvement in cosmetic appearance with better shoulder levelling. The Cavendish grade improved in all patients.

**Conclusion:**

This study emphasizes the results of previous authors demonstrating that CHS can be treated successfully with a VSO. The procedure is simple and its results are reproducible.

## Introduction

The scapula is originally located as a sagittally-oriented structure in the neck. It then migrates to a coronally aligned posterior thoracic position. In congenital high scapula (CHS), failure of of the scapula to descend leaves it in an elevated, malrotated position. The bone is small and broad, the periscapular muscles are hypoplastic and the scapula is rotated with its inferior angle medialised and the glenoid directed inferiorly. Fibrous bands tether the scapula to the chest wall, limiting rotation. The superior angle is attached to the cervical spine by an omovertebral bar (OVB) in 40 % of cases. Associated anomalies of the cervico-thoracic spine are common [1].

Children with CHS have two significant problems: cosmetic and functional due to limited shoulder abduction. Treatment options include simple procedures as excision of the prominent superior angle [2, 3] and major procedures as subtotal scapulectomy [4]. Relocation procedures of the scapula, such as Green’s [5] and Woodward’s [6] procedures, have also been described. Excision of the superomedial angle results only in a cosmetic improvement whereas subtotal scapulectomy is associated with extensive bleeding and a high incidence of brachial plexus injuries. Relocation procedures are associated with lengthy ugly scars, major bleeding, high incidence of brachial plexus palsy and a high incidence of recurrence [1, 7].

Vertical scapular osteotomy (VSO) is another surgical option that can provide both cosmetic and functional improvement. It was originally described by Konig in 1914 [7], then repopularized by Wilkinson and Campbell [7] in 1980. In 2004, MacMurtry et al. [1] reported their experience with this technique in 12 patients.

## Materials and methods

Over a period of 4 years, seven children with CHS were treated at our unit. Three children had associated anomalies of the spine, but none were part of a syndrome. An OVB was present in three patients. The age ranged from 5 to 13 years with an average of 8.4 years. All children were females with unilateral affection (Table 1). We used the Cavendish [3] grading system in our assessment together with measurement of the degree of combined shoulder abduction using a goniometer. Follow up ranged from 2 to 7 years, averaging 4.6 years. All preoperative and post operative assessments were done by the junior author.

**Table 1:** Patients details

Patient #	Age	Gender	OVB	Associations
1	8	F	Y	None
2	9	F	Y	Thoracic scoliosis
3	6	F	N	C3–4 fusion
4	7	F	N	None
5	5	F	Y	T3 HV
6	11	F	N	None
7	13	F	N	None

**Fig. 1:**
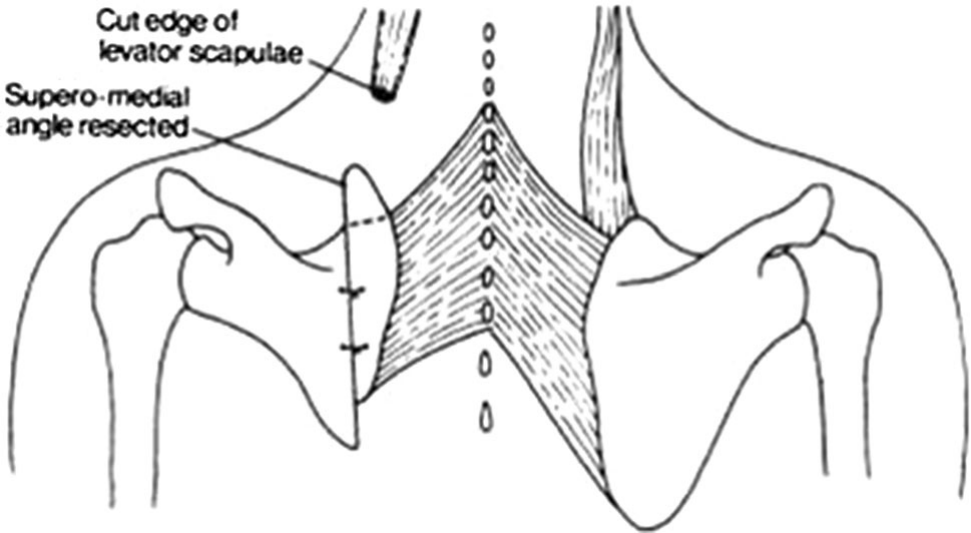
Diagram illustrating the surgical technique of vertical scapular osteotomy [7]

The patient is placed in a semi-prone position on the table with the affected side up and the arm draped separately to allow manipulation of the scapula during the procedure. A vertical incision is made over the scapula, approximately 2 cm lateral to its vertebral border. The approach is deepened through the fascia covering the infraspinatus muscle along the medial border of its origin. The underlying periosteum is incised and is reflected either side to reveal the blade. The line of the osteotomy is marked 1 cm from the vertebral border with the corner of an osteotome. Holes offset at predetermined distances equal to the required degree of displacement are drilled in the bone. The osteotomy is performed using an osteotome, starting at the lower angle and proceeding upwards till the level of the base of the spine. The medial strip is then held with a towel clip and pulled downwards to put tension on the proximal structures. The superior angle of the scapula is separated from all muscle attachments and fibrous bands extraperiosteally, and is then excised using an oscillating saw. The same procedure is performed with the OVB when present. The operation frees the medial strip of the scapula from the major portion of the blade along its whole length. The major portion of the scapula is then held with a towel clip and a finger is swept between the subscapularis and the chest wall in order to locate and divide fibrous bands extending between the bone and the ribs. Both fragments are now mobile and can be realigned in their new position. No attempt is made to bring the inferior angle of the major portion down to the level of the normal side, as the blade is smaller and there is a risk of over-correction, placing traction on the brachial plexus. The aim is to balance the spines of the two scapulae and level the shoulders for cosmetic benefit. Both edges of the scapula are opposed in their new positions and joined together using Ethibond #5 sutures passed through the appropriate drill holes (Fig. 1). Post operatively, a firm pressure dressing is applied and the arm is supported in a sling. Five days after the operation, the dressings are reduced and pendulum movements of the shoulder are encouraged. Physiotherapy is started 6 weeks postoperatively with no restriction on movement.

## Results

**Fig. 2:**
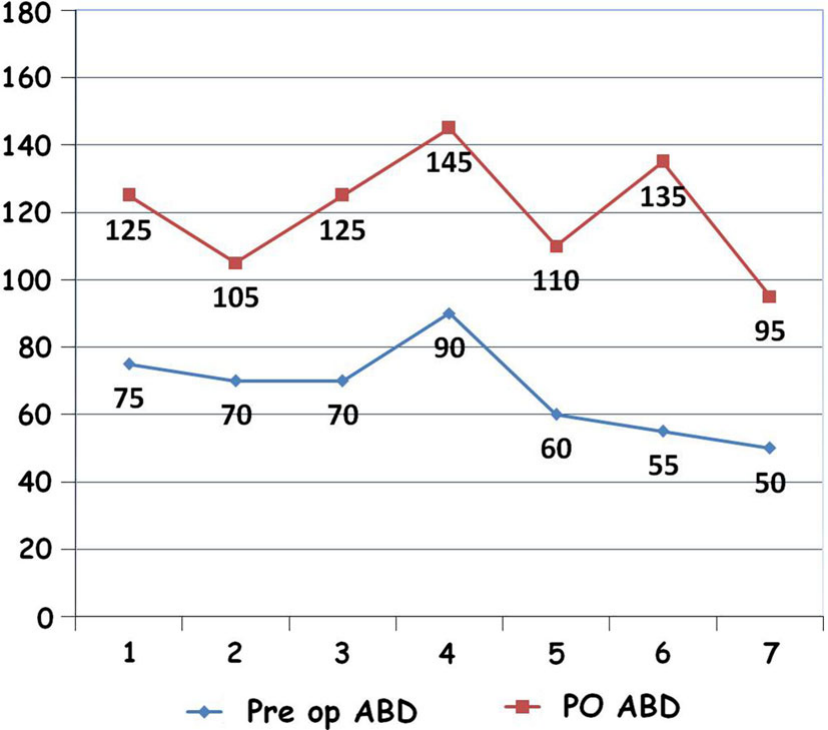
Scatter diagram illustrating the improvement in abduction in each patient

**Fig. 3:**
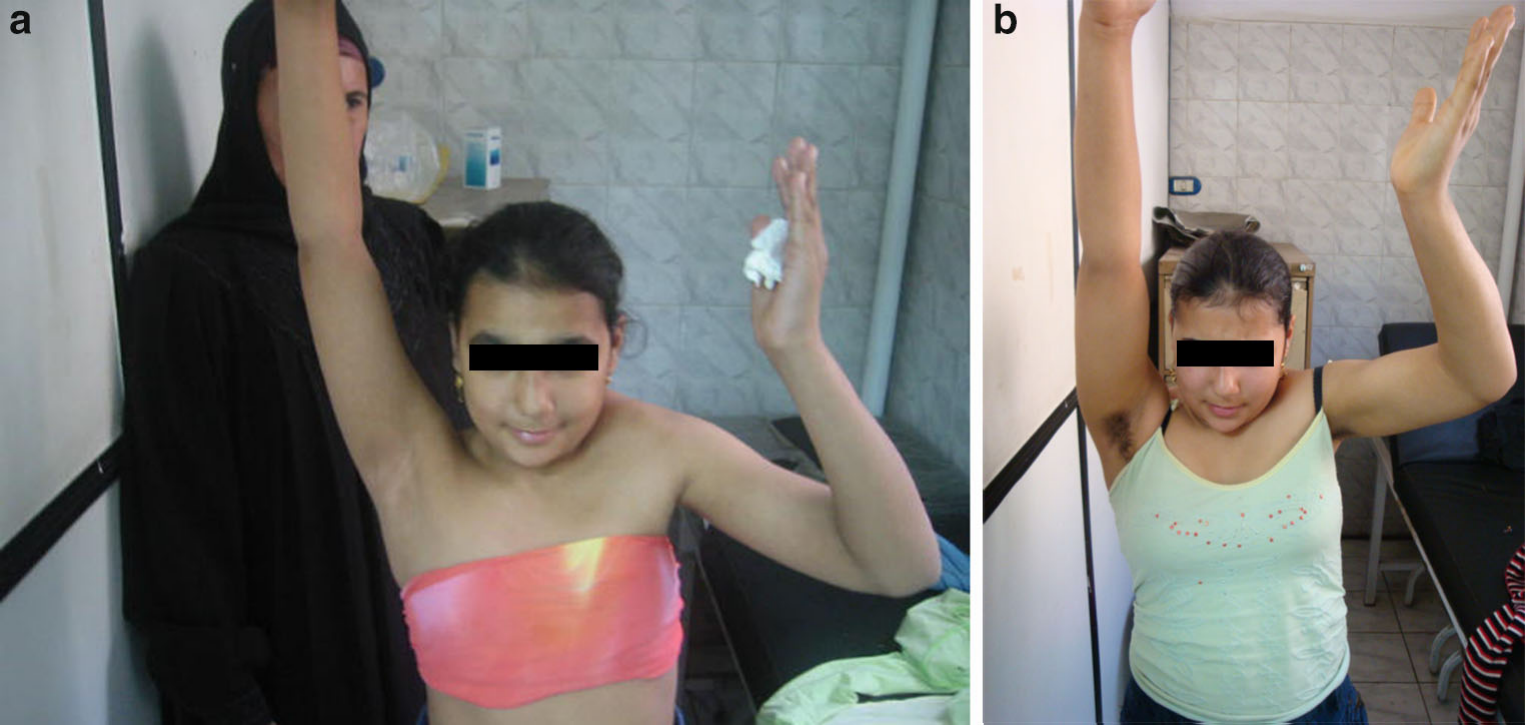
Pre- and post-operative pictures of one girl showing improvement of global abduction from 60° to 110°

All children and parents were extremely satisfied by the results of surgery. Satisfaction was measured by asking the parent(s) to grade their child’s condition at the final follow up on a scale of zero to five; with “zero” meaning totally unsatisfied and “five” meaning extremely satisfied. All patients experienced an improvement in global shoulder abduction (Figs. 2, 3). The mean pre-operative abduction was 67.1° and the mean post-operative (PO) abduction was 120°, with an average gain in abduction of 52.9°.

**Fig. 4:**
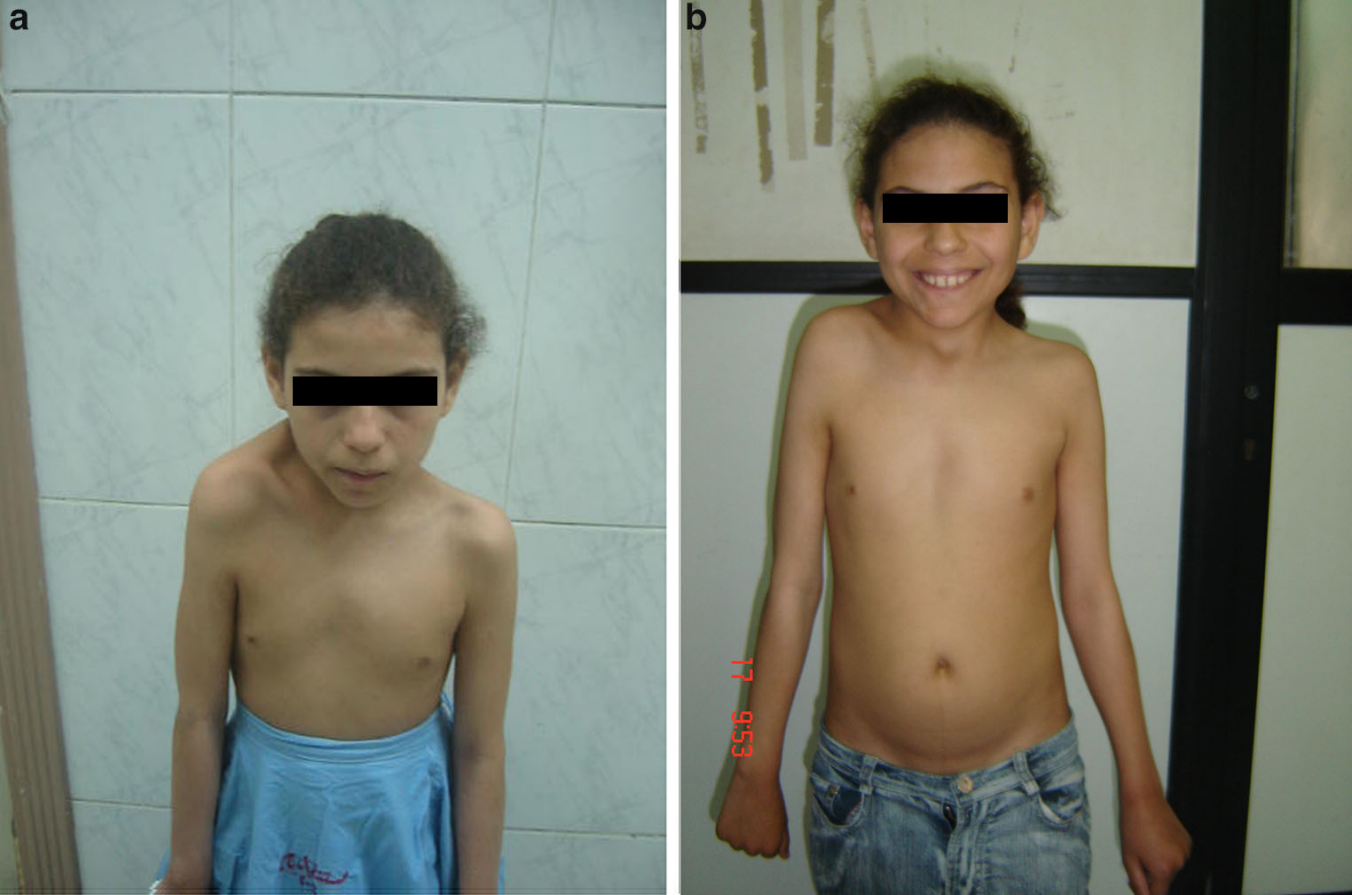
Pre- and post-operative pictures of one girl showing cosmetic improvement with better levelling of the shoulders

**Fig. 5:**
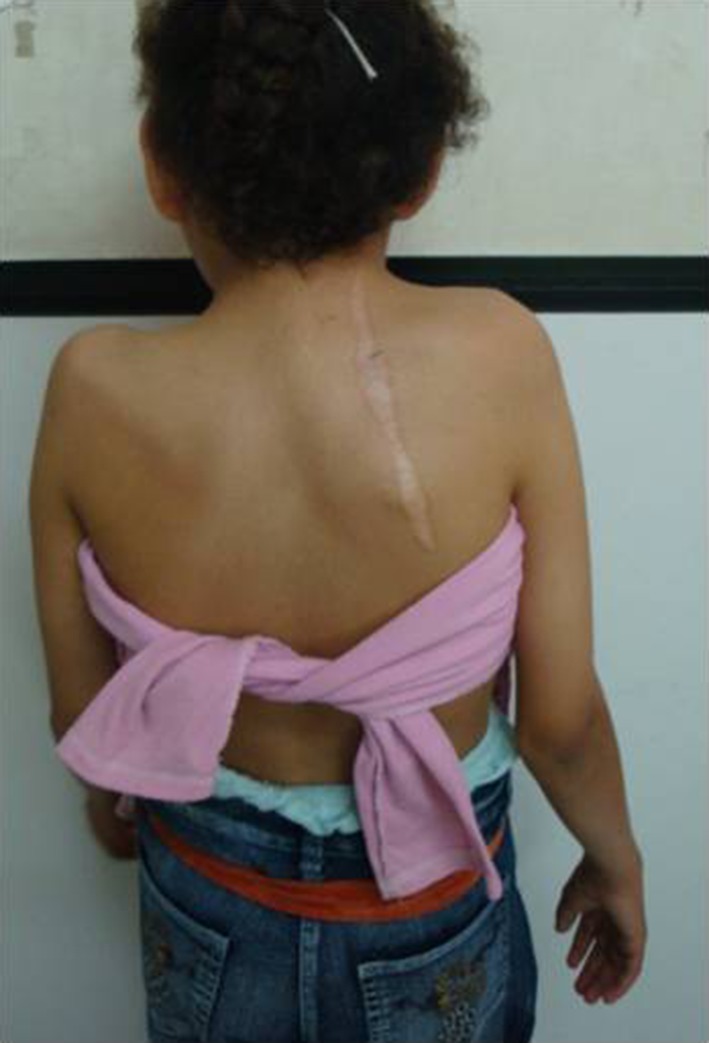
Healing by keloid scar

All patients experienced an improvement in cosmetic appearance with better shoulder levelling (Fig. 4). The Cavendish grade improved in all children; in five patients, it improved two grades, and in two patients, it improved one grade. The average improvement was 1.7 grades. All scars healed by keloid formation and were unsightly; however this was not a source of dissatisfaction for any patient (Fig. 5). No patient developed brachial plexus palsy. The presence of an OVB did not prevent achievement of satisfactory results.

Because CHS is a rare condition, the number of patients included in this study was too small to apply any tests for statistical significance. However, it can be concluded that CHS that is not part of a generalized musculoskeletal syndrome can be treated successfully with VSO regarding both cosmetic and functional aspects.

## Discussion

CHS, also known as Sprengel’s deformity, is a rare congenital anomaly. Patients may have associated anomalies as scoliosis and torticollis and the disease may be part of a syndrome such as Klippel–Feil syndrome. Not every patient who presents with CHS warrants operative treatment; those with a minor cosmetic deformity or minimal functional impairment will not benefit appreciably from operation. The goal is to improve the appearance of the scapula and the neck to the point where the deformity is not noticeable when the patient is dressed; and to increase the combined shoulder abduction where there is significant limitation [8].

The optimal age for operative correction has not been determined. However, most authors recommend that the correction not be performed under 3 years of age. No single procedure can be expected to produce a perfect appearance, particularly in children with associated syndromes such as Klippel–Feil’s syndrome with a short, webbed neck and where rib and chest wall anomalies and scoliosis add to the clinical deformity and are not ameliorated by any procedure that primarily aims to correct only the position of the scapula [8].

Scapular attachments causing limitation of movement are either due to the development of OVB or simple fibrous bands. As noted in the study by Wilkinson and Campbell, improvement in shoulder movement was gained irrespective of the presence of an OVB, indicating that the fibrous bands played a significant role in limiting scapulo-thoracic movements. The fibrous bands run within the rhomboid muscles and when attached to the inferior angle of the scapula, they cause gross malrotation which is easily corrected by their division [7].

This study emphasizes the results of previous authors that CHS can be treated successfully with a VSO. The procedure is simpler than major procedures, such as scapulectomy and scapular relocation procedures, and its results are reproducible. Complications such as brachial plexus injuries and recurrence are much less common, as evidenced from this study and previous reports. The fact that our study did not include any patients with syndromes such as Klippel–Feil’s syndrome may have affected the results. The small number of cases included in the study definitely has its implications on the results, however, it must be appreciated that the condition itself is rare and that several case series studies with small numbers could be included in a future multi-centre study.
