# Erratum to: Vertical scapular osteotomy in congenital high scapula

**DOI:** 10.1007/s11832-015-0686-4

**Published:** 2015-10-01

**Authors:** Tarek Hassan Abdelaziz, Shady Samir

**Affiliations:** Department of Orthopaedic Surgery, Ain Shams University, Cairo, Egypt

## Erratum to: J Child Orthop DOI 10.1007/s11832-015-0676-6

Unfortunately, Figs. 3 and 4 are swapped in the original publication. The correct figures and its relevant captions are given below. The original article is now updated.

**Fig. 3:**
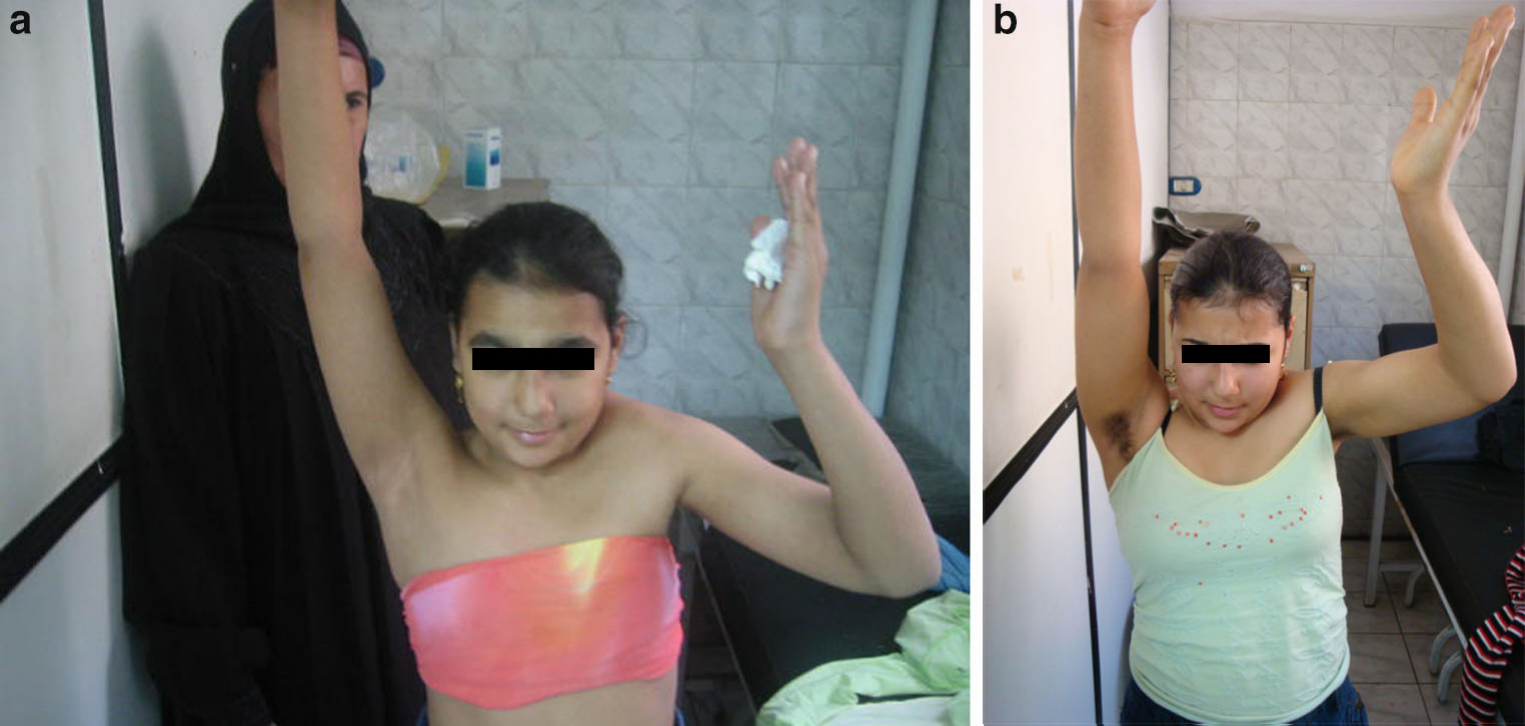
Pre- and post-operative pictures of one girl showing improvement of global abduction from 60° to 110°

**Fig. 4:**
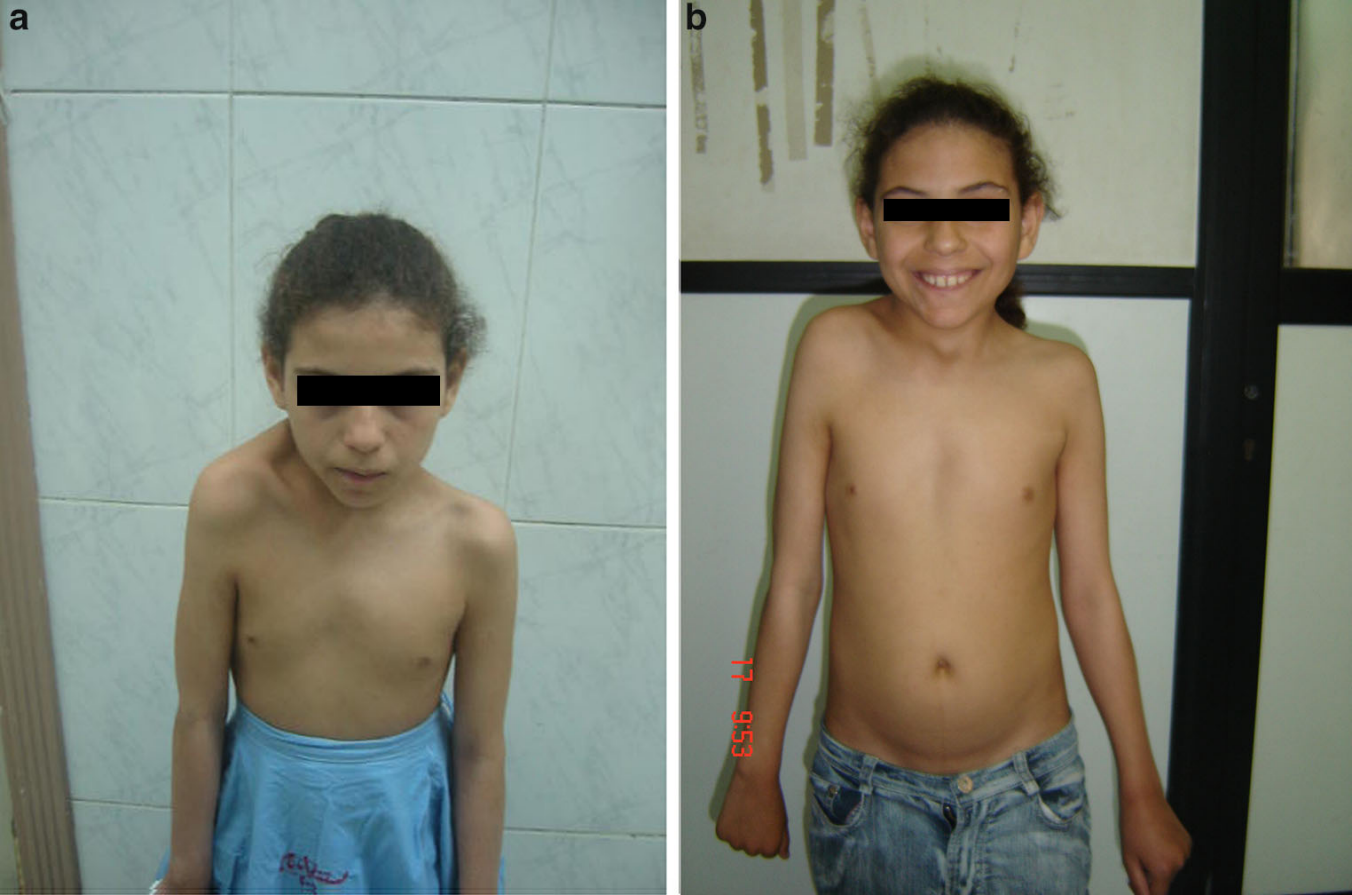
Pre- and post-operative pictures of one girl showing cosmetic improvement with better levelling of the shoulders

